# Dietary Iron Deficiency Impaired Peripheral Immunity but Did Not Alter Brain Microglia in PRRSV-Infected Neonatal Piglets

**DOI:** 10.3389/fimmu.2018.03150

**Published:** 2019-02-04

**Authors:** Brian J. Leyshon, Peng Ji, Megan P. Caputo, Stephanie M. Matt, Rodney W. Johnson

**Affiliations:** ^1^Division of Nutritional Sciences, University of Illinois at Urbana-Champaign, Urbana, IL, United States; ^2^Department of Animal Sciences, University of Illinois at Urbana-Champaign, Urbana, IL, United States; ^3^Neuroscience Program, University of Illinois at Urbana-Champaign, Urbana, IL, United States

**Keywords:** iron, inflammation, microglia, infection, anemia, postnatal, iron deficiency, piglet

## Abstract

During the postnatal period the developing brain is vulnerable to insults including nutrient insufficiency and infection that may lead to disrupted development and cognitive dysfunction. Since iron deficiency (ID) often presents with immunodeficiency, the objective of this study was to investigate peripheral viremia and inflammation as well as brain microglial phenotype and function when ID and respiratory infection occur simultaneously in a neonatal piglet model. On postnatal day 2 (PD 2) male and female piglets were assigned to one of four treatments and fed either control or ID milk replacer. On PD 8 half the pigs on each diet were inoculated with either vehicle or porcine reproductive and respiratory syndrome virus (PRRSV; P-129). Blood samples were collected prior to inoculation (PD 7) and repeated once weekly. Rectal temperature, feeding score, and sickness behavior were measured daily until PD 28. Hematocrit, hemoglobin, and serum iron were reduced by ID but not PRRSV infection. PRRSV-infected piglets displayed viremia by PD 14; however, those fed control diet had lower viral titer on PD 28, while circulating virus remained elevated in those fed an ID diet, suggesting that ID either impaired immune function necessary for viral clearance or increased viral replication. ID piglets infected with PRRSV displayed reduced sickness behavior compared to those fed control diet on PD 13-15 and 18-20. While ID piglet sickness behavior progressively worsened, piglets fed control diet displayed improved sickness score after PD 21. Microglia isolated from PRRSV piglets had increased MHCII expression and phagocytic activity *ex vivo* compared to uninfected piglets. ID did not alter microglial activation or phagocytic activity. Similarly, microglial cytokine expression was increased by PRRSV but unaffected by ID, in stark contrast to peripheral blood mononuclear cell (PBMC) cytokine expression, which was increased by infection and generally decreased by ID. Taken together, these data suggest that ID decreases peripheral immune function leading to increased viremia, but immune activity in the brain is protected from acute ID.

## Introduction

Iron deficiency (ID) and infection often present as comorbid conditions in vulnerable populations, such as infants and young children in developing countries. Respiratory infection is a leading cause of childhood mortality in developing nations and hospitalization for children younger than 1 year old in the United States (1, 2). Iron is important for immune function, and involved with immune cell phagocytic activity (3, 4) and cytokine production (5), with ID contributing to immunocompromise (6, 7). The porcine immune and digestive systems are more similar to humans compared to rodents (8, 9), making pigs an excellent model to study nutrition-immune interactions. Peripheral inflammatory signals may reach the brain through both direct signaling via activation of peripheral afferent nerves, and humoral signaling at the choroid plexus, circumventricular organs, and brain meningeal interface (10, 11). These signals are subsequently amplified and propagated by microglia, leading to a variety of downstream effects, including sickness behavior, a cluster of adaptive behaviors expressed during illness including lethargy, anorexia, and social withdrawal (10). Besides their role as the innate immune cells of the brain, microglia perform neurodevelopmental functions, including synaptic, and dendritic pruning and phagocytosis of neural progenitor cells (12–14). Modulation of microglial activation by peripheral immune signals may alter microglial synaptic pruning and phagocytic activity, adversely affecting neurodevelopment.

The combined effects of ID and infection on neuroinflammation have received little attention, with no postnatal studies to date. Two previous studies used rats to model prenatal ID and postnatal immune challenge with bacterial endotoxin (15, 16). The present study uses the neonatal piglet to model infants developing ID during the early postnatal period and subsequently contracting a respiratory infection, providing a translational model of a serious public health problem—the double burden of early life ID and infection. The first 3 years of life are crucial to brain development, with multiple brain processes including myelination and development of the monoamine neurotransmitter systems beginning prenatally and undergoing the majority of their development postnatally (17). The intricate coordination of multiple neurodevelopmental processes renders the brain vulnerable to environmental insults during this critical period. Individually, both early life ID and postnatal infection are associated with altered neurodevelopment (18–20).

The present study examined the effects and potential interactions of postnatal ID and infection on iron metabolism, peripheral inflammation, neuroinflammation, and microglial phenotype and activity. We hypothesized ID-PRRSV piglets would display a blunted inflammatory cytokine response, impaired ability to clear the infection, and altered iron metabolism in peripheral tissues. Furthermore, we expected microglia from ID infected piglets would be less activated, less phagocytic and would have altered cytokine profiles resulting from blunted peripheral inflammatory cytokine production. Since microglia are key effectors during neurodevelopment, performing functions such as dendritic pruning, understanding their phenotype when confronted with these insults provides useful translational data.

## Materials and Methods

### Animals, Housing, and Experimental Design

A total of 32 crossbred domestic piglets born of dams maintained on an iron-adequate diet throughout gestation were received on postnatal day (PD) 2 from the University of Illinois swine farm. Upon arrival piglets were assigned to one of four treatment groups comprising a 2 × 2 factorial arrangement of diet (control or ID) and infection (control or PRRSV). After controlling for sex, weight, and litter of origin, piglets were distributed into four groups: uninfected and control diet (CN, *n* = 8), uninfected and ID diet (CID, *n* = 8), PRRSV infected and control diet (PN, *n* = 8), and PRRSV infected and ID diet (PID, *n* = 8). Piglets assigned to the control diet received an intramuscular iron dextran injection (200 mg, Uniferon). From PD 2 to 28 piglets were housed individually in cage racks as previously described (21) and were provided a toy (plastic Jingle-Ball; Bio-Serv) for environmental enrichment. Ambient heating was provided to maintain a room temperature of at least 27°C, and supplemental heating was provided by an in-cage heating pad (K&H Lectro-Kennel Heat Pad). A 12 h light/dark cycle was maintained with light from 0700 to 1900 h. All animal care and experimental procedures were performed in accordance with the National Research Council Guide for the Care and Use of Laboratory Animals and approved by the University of Illinois at Urbana-Champaign Institutional Animal Care and Use Committee.

### Diets

Nutritionally complete sow milk replacer diets were formulated by TestDiet (St. Louis, MO) according to previous specifications (21). Piglets were assigned to either an iron-sufficient control diet (100 mg Fe/kg solids) or the ID diet (10 mg Fe/kg solids). Aside from iron levels, all diets met the NRC recommended guidelines (22). Piglets were fed 4 times per day for a total of 300 mL/kg bodyweight. Extra water and swine Blue-Lite (TechMix LLC, Stewart, MN) were provided on a case-by-case basis to piglets per veterinary staff recommendation.

### Inoculation With PRRSV and Daily Assessment of Behavior, Temperature, and Body Weight

On PD 8, piglets were inoculated intranasally with either 1 mL (1 × 10^5^ 50% tissue culture infected dose) of PRRSV (strain P129-BV4, School of Veterinary Medicine at Purdue University, West Lafayette, Indiana) or 1 mL sterile PBS sham as we have described previously (23). PRRSV is a single-stranded RNA virus that produces a chronic interstitial pneumonia in piglets and clinical signs of infection including fever, reduced appetite, and lethargy (24–26). Animals were weighed daily before feeding. Sickness behavior was scored for each piglet upon entering the room and before feeding using a 4-criterion scale quantifying if the piglet displayed lack of appetite, loose stool, panting, or hanging tongue. If a behavior was present, it was marked as a 1; a piglet displaying all 4 behaviors would receive a score of 4, whereas a piglet displaying no sickness behaviors would be scored 0. Rectal temperatures were taken daily. Piglets were assigned a feeding score based on their willingness to complete the first meal of the day (1 = no attempt to eat; 2 = attempted to eat but stopped shortly after; 3 = ate continuously for at least 1 min). PRRSV serum viral load was analyzed by the Veterinary Diagnostic Laboratory via serum multiplex rRT-PCR (University of Illinois, Urbana, IL).

### Assessment of ID and Anemia

Blood samples were taken weekly from the jugular vein using both serum and plasma Vacutainer tubes. An aliquot of whole blood was sent to the University of Illinois Veterinary Diagnostic Laboratory for complete blood count (CBC) with differential staining. The remaining blood was centrifuged at 1,300 × g at 4°C for 15 min to collect serum or plasma and stored at −80°C until analysis. Serum iron was assessed at the University of Illinois Microanalysis Laboratory using inductively-coupled plasma mass spectroscopy (ICP-MS; University of Illinois, Urbana, IL).

### Piglet Necropsy and Brain Dissection

Piglets were sacrificed at PD 28. After anesthetic induction with an intramuscular injection of a telazol:ketamine:xylazine cocktail (50 mg of tiletamine with 50 mg of zolazepam reconstituted with 2.5 mL ketamine (100 g/L) and 2.5 mL xylazine (100 g/L); Fort Dodge Animal Health, Fort Dodge, IA) at a dose of 0.03 mL/kg BW, piglets were sacrificed via intracardiac (i.c.) injection of sodium pentobarbital (86 mg/kg BW; Fatal Plus, Vortech Pharmaceuticals). Blood was collected for CBC, serum, plasma, and PBMC isolation using serum and plasma (EDTA coated) Vacutainer tubes. Liver, spleen, and lung were collected and snap frozen in liquid nitrogen. Piglet brains were removed, weighed, and specific regions of interest were dissected, snap frozen, and stored at −80°C.

### Microglial Isolation

Microglia were isolated from cerebellar tissue based on CD11b expression as previously described (23). Isolation was performed using Miltenyi Biotec GentleMACS C-tubes, Neural Tissue Dissociation Kits (P), GentleMACS Octo Dissociator with Heaters, CD11b MicroBeads, MACS LS columns, and the OctoMACS separator (Miltenyi Biotec, San Diego, CA) according to manufacturer's instructions, with minor modifications. Dissected cerebellar tissue was placed in C-tubes containing Enzyme Mix 1 & 2 (volume doubled from manufacturer specification due to tissue amount). C-tubes were incubated at room temperature for 20 min and attached to the GentleMACS Octo Dissociator with Heaters for mechanical digestion. The tissue homogenate was then forced through a 40 μM Falcon cell strainer to create a single cell suspension. The cell suspension was then added to 30% Percoll PLUS (GE Healthcare, Pittsburgh, PA) and PBS solution and centrifuged to remove myelin. Fc receptor blocking reagent (10 μL/sample) was added to cell suspension and mixed well to prevent non-specific binding to antibodies in the following steps. CD11b MicroBeads were added to the solution and the mixture was then run through MS columns and rinsed, retaining CD11b+ cells in the column while other material passed through. Columns were then removed from the magnet and washed into an Eppendorf tube to obtain the CD11b+ cell fraction, which was centrifuged at 300 × g for 10 min at 4°C to obtain a cell pellet which was immediately used for subsequent assays or stored in TRIzol Reagent (Thermofisher Scientific, Grand Island, NY) at −80°C.

### Flow Cytometry

Cerebellar CD11b+ cells were re-suspended in flow cytometry buffer (1% BSA, 20 mM glucose PBS solution). Cells were stained with CD45 antibody (AbD Serotec, Raleigh, NC) to identify microglia, and MHCII (Antibodies Online, Atlanta, GA) as a marker for immune cell activation. CD11b+ cells expressing low to intermediate levels of CD45 were considered porcine microglia (23). Flow cytometry was performed using a FACS Aria II (BD Biosciences, San Jose, CA). Gating was set according to forward scatter, side scatter, and autofluorescence of an unstained control.

### Microglial Phagocytic Activity

Microglial phagocytic activity was assessed using the Vybrant Phagocytosis Assay Kit (ThermoFisher, Waltham, MA). Cells were diluted to approximately 6 × 10^5^ cells/mL in cell culture medium consisting of DMEM with 10% FBS, and 1% penicillin streptomycin, plated at 5 well-replicates per sample (150 μL/well), and incubated for 24 h at 37°C. After incubation, cell culture medium was replaced with fresh medium. Hundred microliter fluorescein-labeled BioParticles were then added to each well. After incubation for 90 min, the medium was removed by aspiration and 100 μL of 1x trypan blue suspension was added for 10 min at room temperature to quench fluorescence of any remaining BioParticles that were not phagocytosed. The plate was read at an excitation of 485 nm and an emission of 528 nm.

### Quantitative Real-Time PCR

Circulating peripheral blood mononuclear cells (PBMCs) were isolated by Ficoll-Paque Plus gradient according to manufacturer's protocol (GE Healthcare, Pittsburg, PA) and stored in TRIzol Reagent (Thermofisher Scientific, Grand Island, NY) at −80°C until RNA isolation. RNA from microglia, PBMCs, spleen, lung, and liver was isolated using the TRIzol method according to manufacturer protocol. cDNA was synthesized using a High Capacity cDNA Reverse Transcription Kit (Applied Biosystems, Grand Island, NY). Quantitative real-time PCR was performed using the Applied Biosystems Taqman Gene Expression Assay Protocol. Genes of interest related to inflammation, anti-microbial functions, and iron metabolism, were expressed as fold change relative to the endogenous control gene (*RPL19*). Comparison to the endogenous control gene was performed using the 2^−ΔΔCt^ calculation method as previously described (27).

### Statistical Assessment

Statistics were performed in SAS (SAS Institute, Cary, NC) or GraphPad Prism 7 (GraphPad Software Inc., La Jolla, CA). Two piglets in the PID treatment group developed weight loss and hypothermia and were euthanized resulting in *n* = 6. All other piglets completed the study. All data were checked for normality and where applicable, homogeneity of variance. Two-way ANOVA was used to analyze all data, with feeding score, sickness behavior, body weight, temperature, viremia, and CBC measures analyzed as repeated measures using the SAS PROC MIXED function. If significant main effects or interactions were found, Least Significant Difference (LSD) *post-hoc* tests were performed to parse differences between individual groupings. While the current study is not powered to adequately assess sex differences, sex was included in base PROC MIXED analyses. As sex only had significant effects upon temperature, it was not included in subsequent statistical models. Significance is declared at *P* < 0.05.

## Results

### Body Weights, Feeding Score, and Temperature

**Figure 1:**
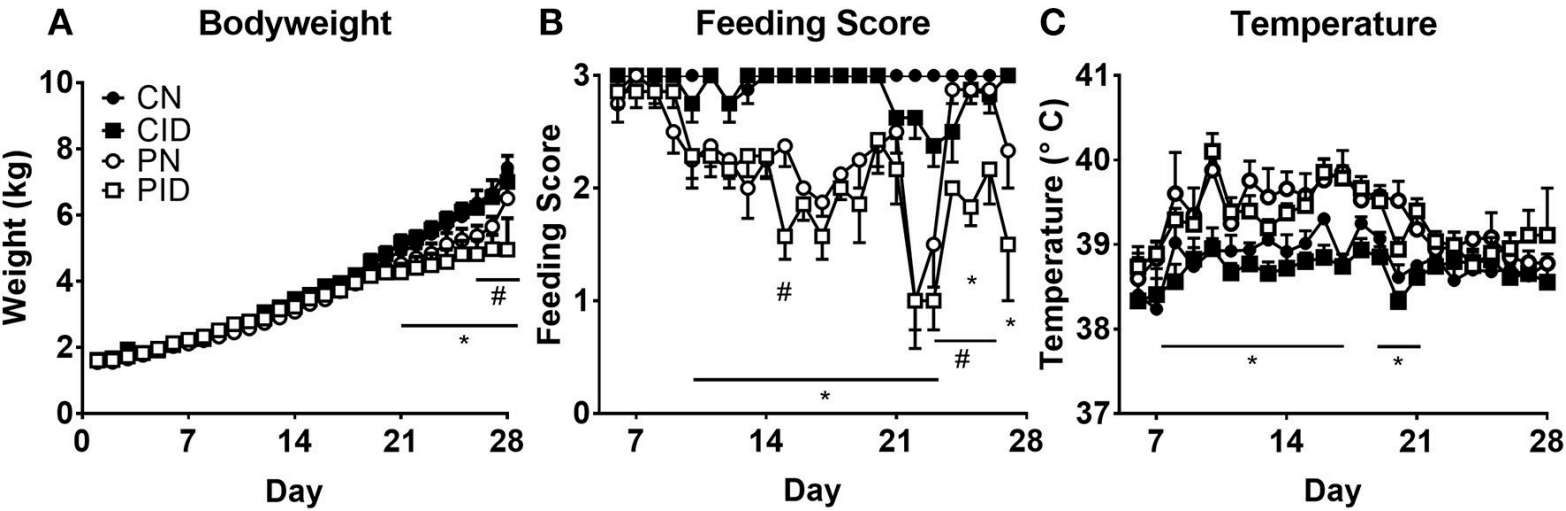
Daily measures of piglet body weights **(A)**, feeding scores **(B)**, and temperature **(C)**. Data are presented as means ± SEM (*n* = 6–8). ^#^denotes differences between dietary groups and ^*^denotes difference between infection groups according to *post-hoc* tests (*p* < 0.05).

Weights were not affected by either infection or diet until day 20 (Figure 1A). Repeated measures ANOVA found several interactions (infection × day, *p* < 0.001; diet × day, *p* = 0.001; infection × diet × day, *p* < 0.001). Two-way ANOVA of PD 27 and PD 28 body weights found effects of infection (*p* = 0.003 and *p* = 0.023, respectively). Repeated measures ANOVA of feeding scores (Figure 1B) found interactions of diet × day (*p* = 0.025) and infection × day (*p* < 0.001). Rectal temperature (Figure 1C) was affected by infection × day (*p* < 0.004).

### Hemoglobin, Hematocrit, and Serum Iron Are Reduced by Diet, but Not Infection

**Figure 2:**
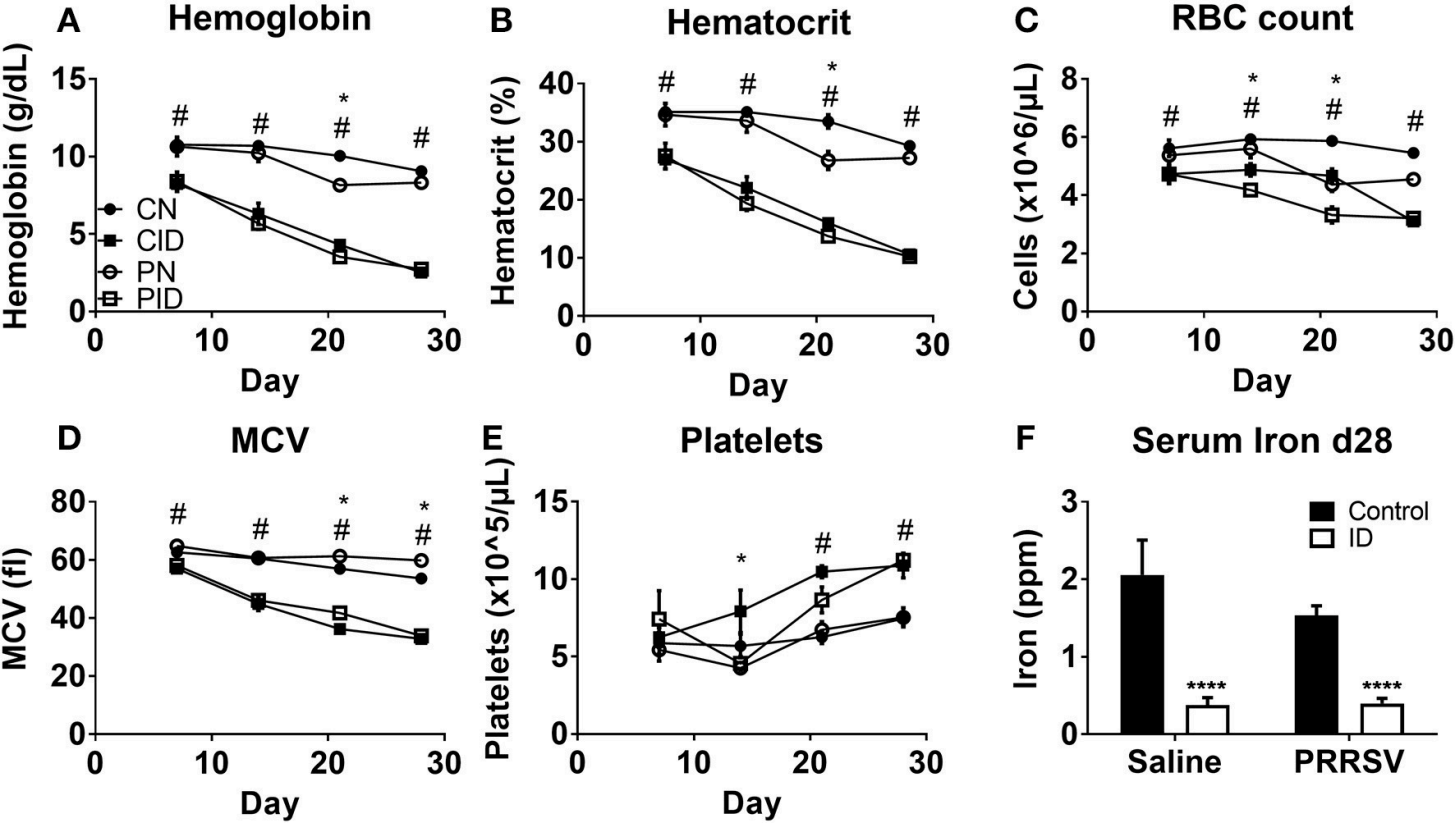
Whole blood hemoglobin **(A)**, hematocrit **(B)**, red blood cell (RBC) count **(C)**, erythrocyte mean cell volume, **(D)**, platelet count **(E)**, at PD 7, 14, 21, and 28, and serum iron at PD 28 **(F)**. Data are presented as means ± SEM (*n* = 6–8). Error bars were not rendered on data points for which the error bars would be shorter than the height of the symbol. ^****^denotes *p* < 0.001. ^#^denotes differences between dietary groups and ^*^denotes difference between infection groups according to *post-hoc* tests (*p* < 0.05).

Piglets fed ID diet developed microcytic anemia. Hemoglobin and hematocrit, standard measures of anemia, were reduced by ID, but not infection (Figures 2A,B). Hemoglobin was affected by interactions between infection × day (*p* < 0.001) and diet × day (*p* < 0.001). *Post-hoc* tests showed PN hemoglobin levels to be different from CN hemoglobin at PD 21 (*p* = 0.001). Hematocrit was reduced by infection × day (*p* = 0.009) and diet × day (*p* < 0.001). As with hemoglobin, PN hematocrit was different from CN values at PD 21 (*p* = 0.001). Red blood cell (RBC) count (Figure 2C) was reduced by diet (*p* < 0.001) and infection × day (*p* = 0.003). PN piglet RBC count decreased on PD 21, and was similar to CID RBC count. By PD 28, PN RBC count increased compared to ID groups but was still reduced compared to controls. This is expected as PRRSV can contribute to anemia (28). Erythrocyte mean cell volume (MCV) was affected by infection (*p* = 0.002) and diet × day (*p* < 0.001). Both ID groups displayed less MCV (Figure 2D). Platelet levels (Figure 2E) were altered by infection × diet × day (*p* = 0.023). Serum iron at PD 28 (Figure 2F) was reduced in ID groups by a main effect of diet (*p* < 0.001). Infection had no effect on serum iron.

### ID Impairs Peripheral Immunity and Viral Clearance

**Figure 3:**
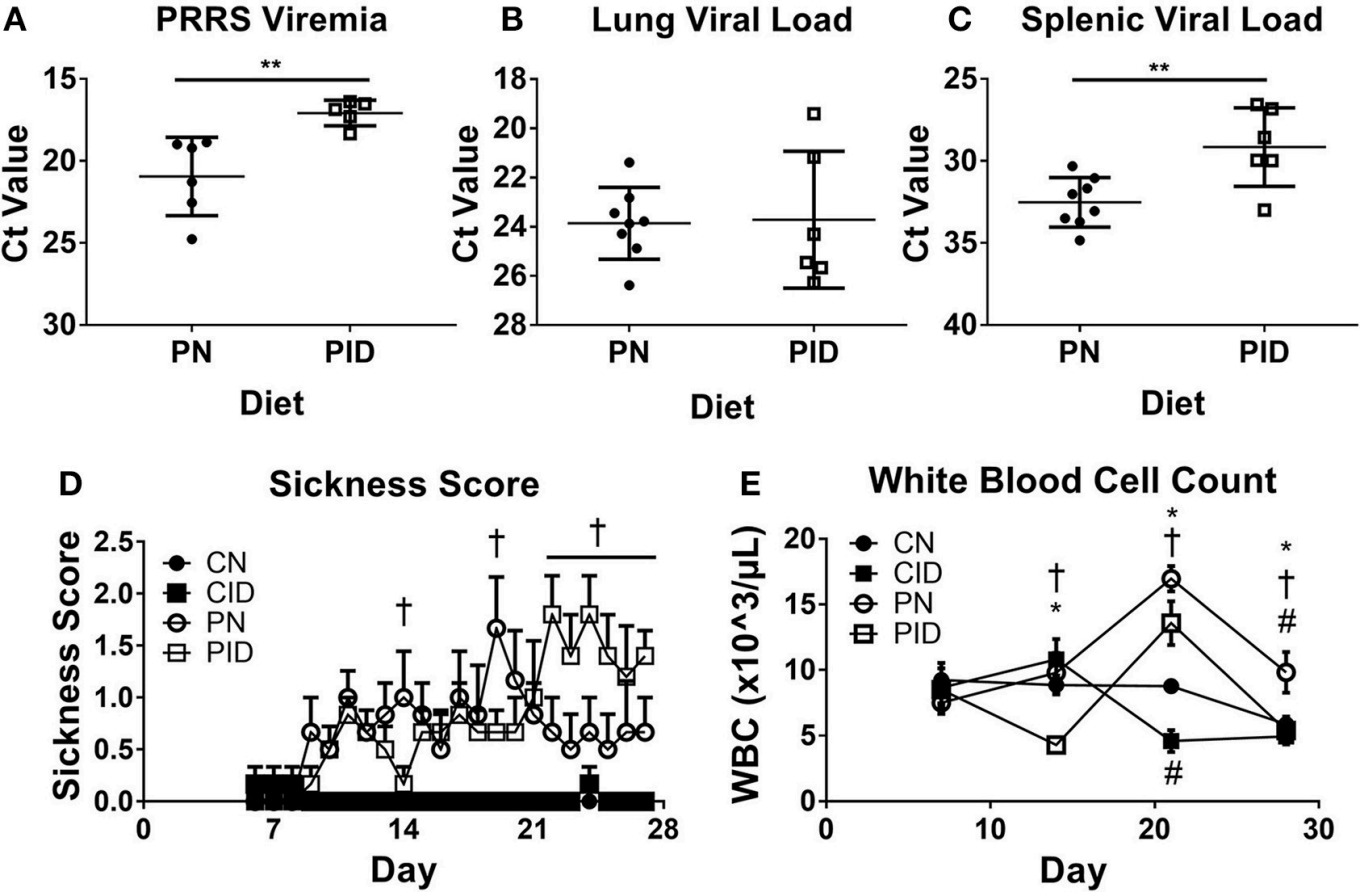
Viremia **(A)**, lung viral load **(B)**, and spleen viral load **(C)** at study termination, sickness score **(D)**, and white blood cell count **(E)**. In **A–C**, the y-axis is inverted to visually convey increased virus, as a lower Ct value indicates higher levels of RNA. Sickness behavior **(D)** was recorded from PD 6 until study termination. White blood cell count **(E)** from weekly blood draws beginning at PD 7 and lasting until study termination. Data are presented as means ± SEM (*n* = 6–8). Error bars were not rendered on data points for which the error bars would be shorter than the height of the symbol. ^**^denotes *p* < 0.01. ^#^denotes differences between dietary groups, ^*^denotes difference between infection groups, and ^†^denotes difference between PN and PID according to *post-hoc* tests (*p* < 0.05).

Viral burden by tissue was assessed by quantifying abundance of PRRSV RNA. Serum viral load, or viremia (Figure 3A), was increased by ID on PD 28 (*p* = 0.007). On PD 14 and 21, there were no differences in viremia between PRRSV-infected groups (not shown). Lung viral load was not affected by dietary group (Figure 3B). However, spleens harvested from PID piglets contained higher quantities of virus than PN spleens (*p* = 0.007, Figure 3C). Sickness behavior (Figure 3D) was affected by interactions between infection × diet × day (*p* = 0.03). Generally, the PID piglets displayed less sickness behavior in the first 3 weeks of the study than the PN piglets, but this pattern inverted after PD 21. During the final week of the study, as PN piglets began to resolve the infection and displayed less sickness behavior, the PID piglets displayed increasingly severe sickness behavior. Analysis of the last week of sickness behavior found main effects of infection (*p* < 0.001) and diet (*p* = 0.041), and a trend for a treatment × diet interaction (*p* = 0.052). White blood cell count (Figure 3E) was altered by interactions of infection × day (*p* < 0.001) and diet × day (*p* = 0.024) were present (Figure 3E). While all groups were similar at the baseline PD 7, PID white blood cell count decreased nearly 50% one week later, while the other groups remained relatively unchanged. ID reduced white blood cell count, which is most clearly illustrated on PD 21. *Post-hoc* tests determined WBC count of each group differed from every other group at PD 21, with each ID group displaying a suppressed WBC count in comparison to its corresponding infection group.

### Both ID and Infection Heavily Modulate PBMC and Liver Expression of Inflammatory, Anti-Microbial, Iron Metabolism, and Especially Anti-Viral Genes

**Figure 4:**
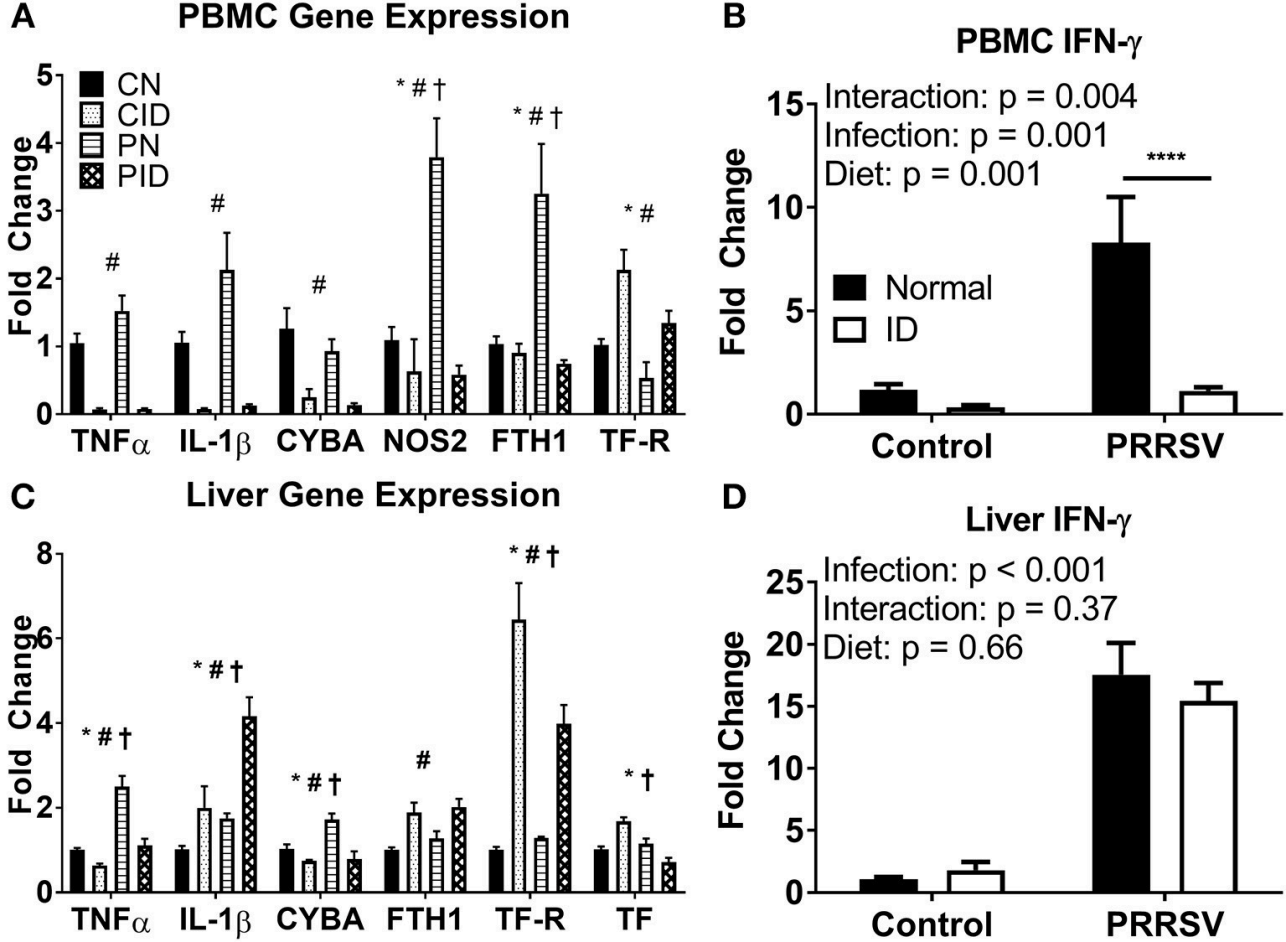
Expression of key genes altered by ID, infection, and their interactions in PBMC **(A,B)** and liver **(C,D)**. For **(A)** and **(C)**, ^*^denotes a main effect of infection, ^#^denotes main effect of diet, and ^†^signifies an interaction between diet and infection. ^****^denotes *p* < 0.001. PBMC, peripheral blood mononuclear cells. Data are presented as means ± SEM (*n* = 6–8).

ID and diet caused widespread changes in peripheral gene expression. Specific genes of interest are highlighted in Figure 4. In PBMCs (Figure 4A), *TNF* and *IL1B* expression were both depressed by ID diet (*p* < 0.001 and *p* < 0.001, respectively). Infection (*p* = 0.06) and infection × diet (*p* = 0.07) trends were present for *TNF*. A similar trend was present for PBMC *IL1B* expression (infection, *p* = 0.06; diet × infection, *p* = 0.087). Liver *TNF* expression was increased in PN, but reduced to baseline by the ID diet, whereas liver *IL1B* expression was increased by ID, with CID and PN *IL1B* expression remaining roughly equal (Figure 4C). PID piglet *IL1B* expression in liver was approximately double that of PN (diet × infection *p* = 0.048). Interferon-γ (IFN-γ) is a key cytokine in the anti-viral response and is imperative for proper immune response against PRRSV infection. *IFNG* expression was heavily upregulated in PBMC of PN piglets (Figure 4B), but reduced to baseline CN expression in PID piglets (*post-hoc* test PN:PID, *p* < 0.001). Effects of diet × infection (*p* = 0.004) modulated PBMC *IFNG* expression. However, only infection influenced liver *IFNG* expression (*p* < 0.001), with no differences due to diet (Figure 4D). Liver expression of *NF-*κ*B*, a key transcription factor in the inflammatory response, was altered by diet × infection (*p* = 0.004); the upregulation of *NF-*κ*B* expression seen in PN piglets was not present in PID piglets. PBMC cytokine expression during infection was depressed by dietary ID, especially production of the antiviral cytokine *IFNG*. Liver cytokine production was differentially modulated by diet during infection, with interactive effects producing both reduced *TNF* expression and increased *IL1B* expression in PID piglets compared to PN piglets, while PID liver *IFNG* expression (Figure 4D) was not depressed by ID as in PBMC (Figure 4B).

Antimicrobial gene expression was differentially altered by PRRSV infection and ID. PBMC expression of *CYBA*, a component of NADPH oxidase, a protein used to generate the macrophage oxidative burst, was downregulated by ID (*p* < 0.001, Figure 4A). Liver *CYBA* expression was altered by diet × infection (*p* = 0.013, Figure 4C). Liver *CYBA* expression was upregulated in PN piglets, but dropped to baseline expression levels in PID piglets. *NOS2*, which also generates reactive oxygen species to fight pathogens, was upregulated during infection. PBMC *NOS2* expression was altered by diet × infection (*p* = 0.001). While PN expression was sharply increased, PID *NOS2* expression was depressed to baseline CN levels by the ID diet. Liver *NOS2* expression was unchanged across all groups. Anti-microbial gene expression is reduced in ID piglets, and the upregulation seen in infected animals fed a normal diet is ablated in infected animals fed an ID diet.

Iron metabolism was profoundly altered by infection. Ferritin (FTH1), the primary intracellular iron storage protein, was sharply upregulated in PBMC of PN piglets, implicating the iron sequestration response to infection. However, ferritin expression in PBMC of PID piglets did not differ from control pigs (Figure 4A). Ferritin expression in PBMC was modulated by diet × infection (*p* = 0.005). Conversely, liver ferritin expression was only altered by diet (*p* = 0.002), and increased by ID diet compared to normal diet (Figure 4C). Transferrin receptor (TFRC) allows cellular uptake of transferrin, and is the primary method by which body cells acquire iron from the circulation. PBMC *TFRC* expression was upregulated by ID diet (main effect, *p* = 0.002), and reduced by infection (main effect, *p* = 0.008). Liver *TFRC* expression was altered by a diet × infection interaction (*p* = 0.013). PN did not differ from CN, though PID displayed an attenuated upregulation compared to CID. Liver transferrin (TF, Figure 4C), the primary chaperone for extracellular iron in the body, showed increased expression in CID piglets, but reduced expression in PID piglets. Both CN and PN groups displayed unchanged expression levels. Effects of diet × infection (*p* < 0.001) altered liver *TF* expression. In PID compared to PN piglets, the iron sequestration response to infection appears to be impaired in PBMC and liver, the two primary depots for iron storage.

### PRRSV Infection and Diet Modulate Gene Expression in PBMCs and the Liver, Though the Lung and Spleen Display Blunted Effects and Are Modulated Only by PRRSV Infection

A complete representation of peripheral genes assessed in this study is presented in Table 1. Diet, PRRSV infection, and diet × infection effects modulated gene expression of PBMC and liver tissue. Conversely, the spleen and lung displayed far fewer effects and were altered only by infection, except for spleen transferrin receptor expression, which was altered by diet × infection (*p* = 0.001). Spleen *TFRC* displayed equivalent upregulation in CID and PN piglets (9 and 12 fold, respectively), while PID spleen *TFRC* was massively upregulated by 50 fold. Generally, inflammatory and iron metabolism gene expression in the spleen and lung were insulated from ID and primarily modulated by PRRSV infection.

**Table 1:** Relative Abundance of mRNA in peripheral tissues^a^.

	**CN**		**CID**		**PN**		**PID**		**Significant effects**		
	**FC**	**SEM**	**FC**	**SEM**	**FC**	**SEM**	**FC**	**SEM**	**PRRSV**	**Diet**	**Interaction**
**PERIPHERAL BLOOD MONONUCLEAR CELLS**											
**Cytokines**											
TNFα	1.05	0.14	0.07	0.02	1.52	0.23	0.08	0.01		^****^	
IL-1β	1.06	0.16	0.08	0.01	2.13	0.55	0.13	0.02		^****^	
IL-6	1.07	0.17	0.19	0.08	3.25	2.30	0.13	0.02			
IL-10	1.07	0.16	0.09	0.02	2.15	0.56	0.17	0.03		^****^	
IFN-γ	1.18	0.28	0.33	0.12	8.30	2.20	1.13	0.18	^***^	^***^	^**^
**Toll-like Receptors**											
TLR3	1.04	0.11	0.30	0.14	0.47	0.12	0.12	0.03	^**^		^****^
TLR4	1.03	0.10	0.08	0.02	1.74	0.86	0.08	0.01		^**^	
TLR7	1.01	0.06	0.09	0.02	2.63	1.31	0.11	0.01		^****^	
**Major Histocompatibility Complex**											
SLA-DRA	1.05	0.15	0.08	0.02	1.39	0.17	0.07	0.01		^****^	
SLA-DRB	1.05	0.14	0.06	0.01	0.93	0.16	0.05	0.01		^****^	
**Anti-Microbial**											
CYBA	1.26	0.30	0.25	0.12	0.93	0.18	0.13	0.03		^****^	
NOS2	1.09	0.19	0.63	0.47	3.79	0.57	0.59	0.13	^**^	^****^	^**^
**Iron Metabolism**											
FTH1	1.03	0.11	0.90	0.14	3.26	0.73	0.75	0.05	^*^	^**^	^**^
FTL	1.00	0.03	2.65	0.29	2.48	0.22	1.68	0.15		^*^	^****^
TFRC	1.02	0.09	2.13	0.30	0.54	0.23	1.34	0.18	^**^	^***^	
HIFα	1.06	0.16	0.45	0.22	0.73	0.15	0.13	0.02	^*^	^***^	
**LIVER**											
**Cytokines**											
TNFα	1.01	0.04	0.64	0.04	2.50	0.25	1.11	0.16	^****^	^****^	^**^
IL-1β	1.02	0.08	2.00	0.51	1.75	0.12	4.16	0.45	^***^	^****^	^*^
IL-4	1.03	0.10	0.47	0.12	0.49	0.12	0.25	0.06	^**^	^**^	
IL-6	1.06	0.18	0.76	0.11	0.88	0.14	0.49	0.05		^*^	
IL-10	1.08	0.20	0.79	0.09	1.81	0.20	0.99	0.09	^**^	^**^	
IFN-γ	1.08	0.19	1.80	0.67	17.54	2.57	15.45	1.44	^****^		
NFκB	1.01	0.07	1.12	0.06	1.49	0.14	1.03	0.04	^*^		^**^
**Toll-like Receptors**											
TLR3	1.02	0.10	0.71	0.12	0.86	0.10	0.97	0.13			
TLR4	1.01	0.05	0.90	0.06	1.21	0.12	0.74	0.13		^**^	
TLR7	1.05	0.17	0.96	0.18	1.81	0.23	0.82	0.12		^**^	^*^
**Major Histocompatibility Complex**											
SLA-DRA	1.01	0.07	0.64	0.07	2.03	0.18	0.95	0.11	^****^	^****^	^**^
SLA-DRB	1.03	0.13	0.59	0.08	1.50	0.19	0.92	0.11	^**^	^**^	
**Anti-Microbial**											
CYBA	1.03	0.11	0.75	0.02	1.73	0.14	0.79	0.18	^**^	^****^	^*^
NOS2	1.03	0.11	0.94	0.13	1.07	0.19	0.79	0.13			
**Iron Metabolism**											
FTH1	1.01	0.06	1.89	0.23	1.28	0.17	2.02	0.19		^***^	
FTL	1.01	0.05	1.38	0.11	1.30	0.08	1.38	0.05		^*^	
TFRC	1.01	0.07	6.44	0.87	1.28	0.04	3.99	0.44	^*^	^****^	^*^
TF	1.01	0.07	1.68	0.09	1.15	0.12	0.71	0.11	^***^		^****^
HIFα	1.04	0.12	0.97	0.07	1.00	0.14	0.95	0.07			
**Acute Phase Response Proteins**											
HAMP	1.38	0.44	0.00	0.00	0.65	0.23	0.01	0.00		^**^	
CRP	1.32	0.45	0.60	0.11	3.87	1.84	4.06	1.67	^*^		
**SPLEEN**											
**Cytokines**											
TNFα	1.09	0.15	0.78	0.15	0.81	0.10	0.60	0.09			
IL-1β	1.35	0.40	1.26	0.19	1.74	0.33	2.73	0.43	^*^		
IL-6	1.07	0.16	0.70	0.15	0.68	0.06	0.76	0.14			
IL-10	1.17	0.28	0.69	0.13	0.81	0.06	0.72	0.07			
IFN-γ	1.06	0.13	0.99	0.14	6.86	1.35	10.04	1.24	^****^		
NFκB	1.04	0.11	1.06	0.11	1.01	0.11	0.84	0.08			
**Toll-like Receptors**											
TLR3	1.33	0.34	0.88	0.29	3.10	1.31	1.61	0.46			
TLR4	1.03	0.08	0.95	0.16	0.77	0.10	0.72	0.09	^*^		
TLR7	1.02	0.08	0.68	0.10	0.99	0.09	0.75	0.12	^**^		
**Anti-Microbial**											
CYBA	1.21	0.24	0.86	0.16	3.02	1.23	1.52	0.62			
NOS2	1.95	0.48	1.58	0.48	2.45	1.22	1.36	0.62			
**Iron Metabolism**											
FTH1	1.03	0.10	1.30	0.20	3.86	1.67	1.98	0.25			
FTL	1.12	0.24	0.84	0.09	2.62	1.06	1.94	0.40	^*^		
TFRC	1.27	0.32	8.90	4.32	12.15	1.78	69.60	12.45	^****^	^****^	^***^
HIFα	1.13	0.18	0.84	0.15	2.03	1.14	1.30	0.27			
**LUNG**											
**Cytokines**											
TNFα	1.03	0.10	2.07	0.65	3.65	1.17	2.42	0.59			
IL-1β	1.24	0.35	1.21	0.26	9.18	3.37	8.04	2.34	^**^		
IL-4	1.05	0.13	0.65	0.17	1.23	0.46	0.76	0.26			
IL-6	1.10	0.19	1.16	0.17	0.83	0.24	1.49	0.78			
IL-10	1.03	0.10	1.67	0.36	2.15	0.63	2.81	0.61			
IFN-γ	1.05	0.11	2.04	0.63	24.13	10.36	20.22	5.80	^**^		
NFκB	1.03	0.10	1.20	0.14	1.63	0.21	1.69	0.33	^*^		
**Toll-like Receptors**											
TLR3	1.29	0.29	0.64	0.25	2.42	1.07	1.27	0.38			
TLR4	1.00	0.07	1.19	0.17	1.90	0.65	1.32	0.20			
TLR7	1.05	0.13	1.28	0.22	1.75	0.32	2.02	0.34	^*^		
**Major Histocompatibility Complex**											
SLA-DRA	1.03	0.11	1.31	0.25	2.89	0.28	2.49	0.25	^****^		
SLA-DRB	1.04	0.11	1.32	0.33	1.80	0.23	1.94	0.28	^*^		
**Anti-Microbial**											
CYBA	1.31	0.33	1.04	0.25	3.01	1.14	1.50	0.57			
NOS2	1.04	0.12	1.14	0.13	1.05	0.50	0.62	0.24			
**Iron Metabolism**											
FTH1	1.03	0.10	1.15	0.11	4.16	1.70	2.02	0.25	^*^		
FTL	1.01	0.07	0.95	0.07	3.30	1.32	2.47	0.48	^*^		
TFRC	1.19	0.26	1.85	0.79	3.00	0.63	4.84	1.51	^**^		
HIFα	1.15	0.20	0.71	0.13	2.01	1.05	1.19	0.26			

a Statistical effects determined by 2-way ANOVA.

* signifies p < 0.05,

** p < 0.01,

*** p < 0.001,

**** *p < 0.001. CN, control, normal diet; CID, control, ID diet; FC, fold change; PN, PRRSV-infected, normal diet; PID, PRRSV infected, ID diet*.

### Microglial Gene Expression Is Altered by Infection, but Insulated From the Effects of ID

**Figure 5:**
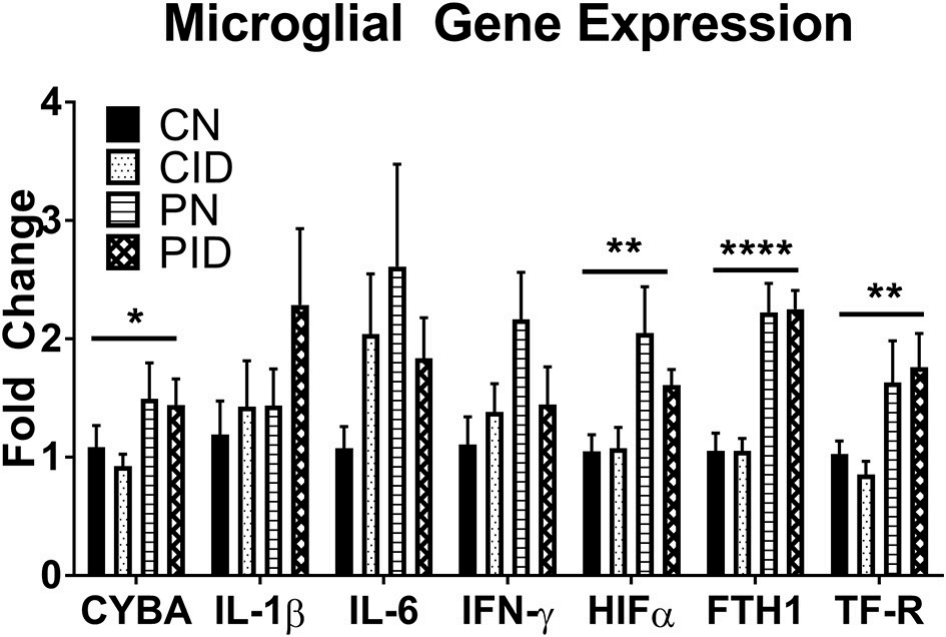
Microglial gene expression. Data are presented as means ± SEM (*n* = 6–8). Asterisks denote main effect of PRRSV infection, ^*^p < 0.05, ^**^p < 0.01, ^****^p < 0.001.

Microglial expression of anti-microbial and iron metabolism genes was altered by PRRSV infection, but not ID (Figure 5). Infection increased *CYBA* expression (main effect, *p* = 0.043). Expression of *HIF1A* was increased by infection (main effect, *p* = 0.041). HIFα is a transcription factor involved in regulating cellular responses to infection and hypoxia and modulates many cellular processes, including angiogenesis, cell proliferation, and energy and iron metabolism. Ferritin and transferrin receptor expression were upregulated by infection (main effects, *p* < 0.001 and *p* = 0.005, respectively). Anti-microbial and iron metabolism genes were upregulated by infection, but microglial gene expression appears largely insulated from the effects of ID.

### Infection, but not ID, Alters Microglial Activation, and Phagocytic Activity

**Figure 6:**
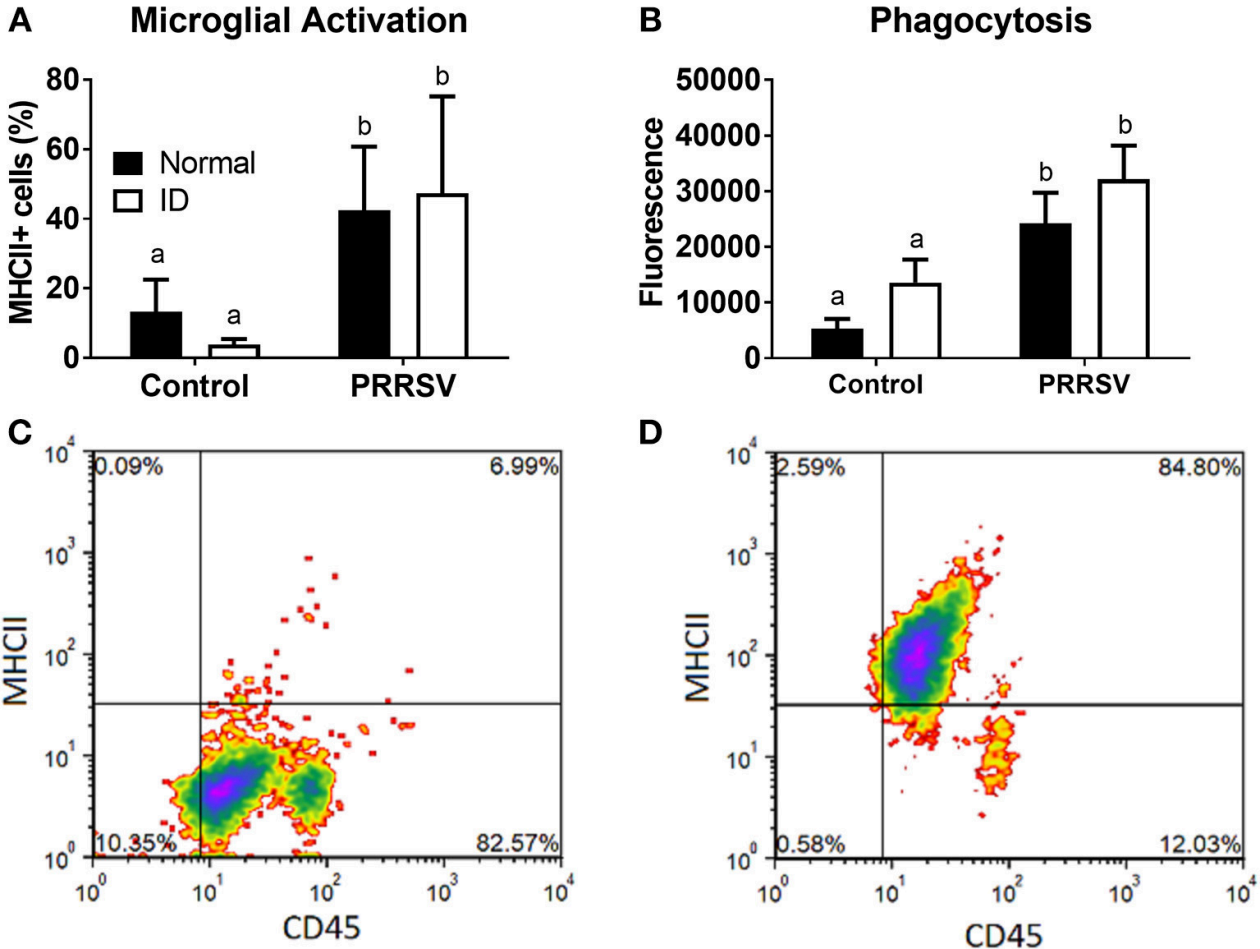
Microglial activation **(A)** and phagocytic activity **(B)**. Cells positive for CD45 and MHCII were considered activated. Representative density plots of CD11b+ cells from an uninfected piglet **(C)** and CD11b+ cells from a PRRSV-infected piglet **(D)**. Lower case letters denote significantly different groups as determined by Fisher's LSD (*p* < 0.05). Data are presented as means ± SEM (*n* = 6–8).

Flow cytometry and measurement of phagocytic activity were used to assess microglial phenotype. CD11b+ cells expressing both CD45 and MHCII were considered activated microglia. Percentage of activated microglia was averaged for each group (Figure 6A). A two-way ANOVA found a main effect of infection (*p* < 0.001); no other effects were found. Representative plots of control and PRRSV-infected piglet microglia are depicted by Figures 6C,D, respectively. The percentage of CD45+ and MHCII+ cells are presented in the upper-right corner of the representative plots. Microglial phagocytosis (Figure 6B) was assessed using the Vybrant Phagocytosis Assay Kit. A main effect of infection (*p* = 0.002) increased phagocytic activity. No other effects were present, and dietary groups did not differ. Lower case letters denote significantly different groups as determined by Fisher's LSD (*p* < 0.05). Data are presented as means ± SEM (*n* = 6–8).

## Discussion

The present study was designed to evaluate the dual burden of ID and infection on several indices of immune activation in the periphery and brain. ID is the most common micronutrient deficiency in the world (29). As ID often presents with immunodeficiency, the double burden of ID and infection in early life is of serious concern, especially in developing nations. Though there is evidence ID alters immune cell function (3, 5, 6), more work is required to determine specifically how this leads to immunocompromise. To date, there has been no work studying the interactions of ID and infection upon neuroinflammation in the postnatal period. To address this question, the current work used a translational neonatal piglet model raised to a developmental time point analogous to that of a 4–6 month old human infant (30).

Our data show that while infection did not alter measures of anemia in the context of ID, ID reduced viral clearance from both the serum and spleen, suggesting a direct effect of ID upon immune cells, thereby reducing their efficacy. White blood cell counts were reduced in ID piglets compared to piglets fed normal diet; reduced immune cell proliferation may be one route by which ID weakens the immune system. Interestingly, PID piglets displayed a pattern of sickness behavior largely inverse to that of infected piglets fed a normal diet (Figure 3). PID piglets demonstrated fewer stereotypical sickness behaviors during the first 3 weeks of the study, displaying greater appetite and activity levels compared to PN piglets. However, this phenomenon inverted during the last week of the study, wherein the condition of PID piglets sharply declined. During this final week, as PN piglets were recovering steadily, PID piglets displayed a sharp increase in sickness behaviors which persisted to the end of the study. While there are examples of nutrition attenuating the sickness behavior response (31), there is little data to be found on malnutrition exacerbating sickness behavior. The public health impact of ID upon bacterial and viral infection should not be underestimated and requires further study.

There are several possible mechanisms which may contribute to the atypical illness progression observed in PRRSV piglets. The heightened viral load compared to piglets fed a normal diet indicates impaired immune function and may cause comparatively increased tissue damage, contributing to the observed decline in PID piglet condition. ID had marked effects on PBMC cytokine expression, reducing TNFα, IL-1β, IFN-γ, and IL-10. While the first three pro-inflammatory cytokines promote the sequestration of iron by immune cells, the anti-inflammatory cytokine IL-10 promotes resolution of inflammation and also promotes iron export from immune cells, reversing the iron sequestration response to inflammation (32). The generally reduced cytokine expression in PID piglets compared to PN piglets indicates a blunted infection response. Changes in IFN-γ expression, which was reduced to basal levels in PID piglets compared to an 8-fold increase in PN piglets, are notable as IFN-γ is a key mediator in antiviral immunity and imperative for clearance of the PRRS virus (33). T-cells are the primary producers of IFN-γ and are PMBCs. Interestingly, directional changes in expression differed across tissues - while IL-1β expression was decreased in PID PBMCs, it increased in the liver. PBMC expression of Toll-like Receptors 3 and 7, which detect double- and single-stranded RNA, respectively, was reduced by ID, which may further depress the immune response to viral infection. ID reduced the antimicrobial capacity of PBMCs, as expression of both NADPH oxidase and NOS2, which are reliant upon iron to generate reactive oxygen species to destroy pathogens, was reduced, matching previous findings showing iron chelation reduced iNOS activity in a murine macrophage cell line (34). Thus, ID impairs both the initial inflammatory response to infection as well as subsequent resolution of the inflammatory response in PBMCs as well as liver tissue. These data are valuable as the porcine immune system is highly similar to the human immune system (9), and the pig has functional equivalents to all cells and cytokines involved in the Th1/Th2/Th17/Treg axis (35).

The brain is sensitive to peripheral inflammatory signals, which promote neuroinflammation, activation of microglia, and sickness behavior (36). During neurodevelopment, microglia perform synaptic pruning and phagocytosis of neural precursor cells (13, 14), which may be modulated by early life environmental insults. While microglial activation and phagocytic activity were increased due to postnatal infection in agreement with previous results (23), ID did not alter microglial activation or phagocytic activity. Microglial gene expression was also altered by infection alone, with no diet effects. Cytokine and antimicrobial gene expression were increased by infection, as were genes involved in iron sequestration, suggesting microglia were insulated from the effects of iron deficiency (ID), at least at the observed time point. The brain is “iron privileged” compared to the periphery, e.g., in the case of deficiency, the brain is depleted of iron more slowly, though red blood cells are prioritized over the brain to receive iron. Therefore, in periods of negative iron balance, such as increased erythropoiesis associated with development concurrent with insufficient nutritional intake, the brain is vulnerable to ID (37). Previous data from our lab found hippocampal iron content from ID piglets showed significantly reduced iron levels, though iron levels in cortical tissue did not differ from those of controls (38). In the current study, microglial phenotype and cytokine expression may be unaffected as brain tissue is still prioritized over peripheral tissues such as skeletal muscle and heart (37), thus iron levels in microglia may not have reached the threshold for dysfunction compared to peripheral immune cells. Therefore, the altered sickness behavior observed in PID piglets likely results from the altered expression of cytokines in the periphery. It is possible that continued ID would eventually affect microglial physiology.

To our best knowledge, this is one of the first studies to investigate how postnatal ID in the context of infection affects microglia and neuroinflammation. Despite drastically altered sickness behavior and abundant differences in the peripheral immune response of PID piglets, microglial activation state, activity, and cytokine expression were unaffected by ID. Thus, at the time point assessed in this study, microglia do not appear to be affected by ID, though further study is required. ID is a major global health concern, and these data demonstrate the egregious impact nutritional ID has upon immunity in the postnatal period, highlighting the importance of public health efforts to reduce the prevalence of ID in early life with its associated increased susceptibility to infection.

## Author Contributions

RJ and BL designed the study. BL, PJ, MC, SM conducted research. BL, MC, SM analyzed data. BL, PJ, MC, SM, RJ wrote the paper. RJ had primary responsibility for final content. All authors read and approved the final manuscript.

### Conflict of Interest Statement

The authors declare that the research was conducted in the absence of any commercial or financial relationships that could be construed as a potential conflict of interest.

## References

[1] Lim SS Vos T Flaxman AD Danaei G Shibuya K Adair-Rohani H. A comparative risk assessment of burden of disease and injury attributable to 67 risk factors and risk factor clusters in 21 regions, 1990–2010: a systematic analysis for the Global Burden of Disease Study 2010. Lancet (2012) 380:2224–60. 10.1016/S0140-6736(12)61766-8 23245609 PMC4156511

[2] Ferkol T Schraufnagel D. The global burden of respiratory disease. Ann Am Thorac Soc. (2014) 11:404–6. 10.1513/AnnalsATS.201311-405PS 24673696

[3] Bergman M Salman H Pinchasi R Straussberg R Djaldetti M Bessler H. Phagocytic capacity and apoptosis of peripheral blood cells from patients with iron deficiency anemia. Biomed Pharmacother. (2005) 59:307–11. 10.1016/j.biopha.2004.11.009 15996848

[4] Nairz M Schroll A Demetz E Tancevski I Theurl I Weiss G. 'Ride on the ferrous wheel'–the cycle of iron in macrophages in health and disease. Immunobiology (2015) 220:280–94. 10.1016/j.imbio.2014.09.010 25240631

[5] Bergman M Bessler H Salman H Siomin D Straussberg R Djaldetti M. In vitro cytokine production in patients with iron deficiency anemia. Clin Immunol. (2004) 113:340–4. 10.1016/j.clim.2004.08.011 15507399

[6] Golz A Netzer A Goldenberg D Westerman ST Westerman LM Joachims HZ. The association between iron-deficiency anemia and recurrent acute otitis media. Am J Otolaryngol. (2001) 22:391–4. 10.1053/ajot.2001.28075 11713723

[7] Jason J Archibald LK Nwanyanwu OC Bell M Jensen RJ Gunter E The effects of iron deficiency on lymphocyte cytokine production and activation: preservation of hepatic iron but not at all cost. Clin Exp Immunol. (2001) 126:466–73. 10.1046/j.1365-2249.2001.01707.x 11737064 PMC1906222

[8] Schook L Beattie C Beever J Donovan S Jamison R Zuckermann F. Swine in biomedical research: creating the building blocks of animal models. Anim. Biotechnol. (2005) 16:183–90. 10.1080/10495390500265034 16342425

[9] Mair KH Sedlak C Käser T Pasternak A Levast B Gerner W. The porcine innate immune system: an update. Dev Comparat Immunol. (2014) 45:321–43. 10.1016/j.dci.2014.03.022 24709051 PMC7103209

[10] Dantzer R O'Connor JC Freund GG Johnson RW Kelley KW. From inflammation to sickness and depression: when the immune system subjugates the brain. Nat Rev Neurosci. (2008) 9:46–56. 10.1038/nrn2297 18073775 PMC2919277

[11] Louveau A Plog BA Antila S Alitalo K Nedergaard M Kipnis J. Understanding the functions and relationships of the glymphatic system and meningeal lymphatics. J Clin Invest. (2017) 127:3210–9. 10.1172/JCI90603 28862640 PMC5669566

[12] Cunningham CL Martínez-Cerdeño V Noctor SC. Microglia regulate the number of neural precursor cells in the developing cerebral cortex. J Neurosci. (2013) 33:4216–4233. 10.1523/JNEUROSCI.3441-12.2013 23467340 PMC3711552

[13] Sierra A Abiega O Shahraz A Neumann H. Janus-faced microglia: beneficial and detrimental consequences of microglial phagocytosis. Front Cell Neurosci. (2013) 7:6. 10.3389/fncel.2013.00006 23386811 PMC3558702

[14] Gomez-Nicola D Perry VH. Microglial dynamics and role in the healthy and diseased brain: a paradigm of functional plasticity. Neuroscientist (2015) 21:169–84. 10.1177/1073858414530512 24722525 PMC4412879

[15] Harvey L Boksa P. Do prenatal immune activation and maternal iron deficiency interact to affect neurodevelopment and early behavior in rat offspring? Brain Behav Immun. (2014) 35:144–54. 10.1016/j.bbi.2013.09.009 24064370

[16] Harvey L Boksa P. Additive effects of maternal iron deficiency and prenatal immune activation on adult behaviors in rat offspring. Brain Behav Immun. (2014) 40:27–37. 10.1016/j.bbi.2014.06.005 24930842

[17] Cusick SE Georgieff MK The role of nutrition in brain development: the golden opportunity of the “First 1000 Days.” J Pediatr. (2016) 175:16–21. 10.1016/j.jpeds.2016.05.013 27266965 PMC4981537

[18] Lozoff B Beard J Connor J Barbara F Georgieff M Schallert T. Long-lasting neural and behavioral effects of iron deficiency in infancy. Nutr Rev. (2006) 64:S34–43. 10.1301/nr.2006.may.S34-S43 16770951 PMC1540447

[19] Beard JL. Why iron deficiency is important in infant development. J Nutr. (2008) 138:2534–6. 10.1093/jn/138.12.2534 19022985 PMC3415871

[20] Conrad MS Harasim S Rhodes JS Van Alstine WG Johnson RW. Early postnatal respiratory viral infection alters hippocampal neurogenesis, cell fate, and neuron morphology in the neonatal piglet. Brain Behav Immun. (2015) 44:82–90. 10.1016/j.bbi.2014.08.009 25176574 PMC4275372

[21] Leyshon BJ Radlowski EC Mudd AT Steelman AJ Johnson RW. Postnatal iron deficiency alters brain development in piglets. J Nutr. (2016) 146:1420–7. 10.3945/jn.115.223636 27281804 PMC4926848

[22] Council NR Nutrient Requirements of Swine: 10th Revised Edition. Washington, DC: The National Academies Press (1998).

[23] Ji P Schachtschneider KM Schook LB Walker FR Johnson RW. Peripheral viral infection induced microglial sensome genes and enhanced microglial cell activity in the hippocampus of neonatal piglets. Brain Behav Immun. (2016) 54:243–51. 10.1016/j.bbi.2016.02.010 26872419 PMC4828316

[24] Duan X Nauwynck HJ Pensaert MB. Virus quantification and identification of cellular targets in the lungs and lymphoid tissues of pigs at different time intervals after inoculation with porcine reproductive and respiratory syndrome virus (PRRSV). Vet Microbiol. (1997) 56:9–19. 10.1016/S0378-1135(96)01347-8 9228678

[25] Chen XX Quan R Guo XK Gao L Shi J Feng WH. Up-regulation of pro-inflammatory factors by HP-PRRSV infection in microglia: implications for HP-PRRSV neuropathogenesis. Vet Microbiol. (2014) 170:48–57. 10.1016/j.vetmic.2014.01.031 24581811

[26] Elmore MR Burton MD Conrad MS Rytych JL Van Alstine WG Johnson RW. Respiratory viral infection in neonatal piglets causes marked microglia activation in the hippocampus and deficits in spatial learning. J Neurosci. (2014) 34:2120–9. 10.1523/JNEUROSCI.2180-13.2014 24501353 PMC3913866

[27] Livak KJ Schmittgen TD. Analysis of relative gene expression data using real-time quantitative PCR and the 2(-Delta Delta C(T)) method. Methods (2001) 25:402–8. 10.1006/meth.2001.1262 11846609

[28] Halbur RG Pallares FJ Rathje JA Evans R Hagemoser WA Paul PS. Effects of different us isolates of porcine reproductive and respiratory syndrome virus (PRRSV) on blood and bone marrow parameters of experimentally infected pigs. Vet Rec. (2002) 151:344–8. 10.1136/vr.151.12.344 12371690

[29] Fretham SJ Carlson ES Georgieff MK. The role of iron in learning and memory. Adv Nutr. (2011) 2:112–21. 10.3945/an.110.000190 22332040 PMC3065765

[30] Conrad MS Dilger RN Johnson RW. Brain growth of the domestic pig (Sus scrofa) from 2 to 24 weeks of age: a longitudinal MRI study. Dev Neurosci. (2012) 34:291–8. 10.1159/000339311 22777003 PMC3646377

[31] Clouard C Souza AS Gerrits WJ Hovenier R Lammers A Bolhuis JE. Maternal fish oil supplementation affects the social behavior, brain fatty acid profile, and sickness response of piglets. J Nutr. (2015) 145:2176–84. 10.3945/jn.115.214650 26180250

[32] Nairz M Haschka D Demetz E Weiss G. Iron at the interface of immunity and infection. Front Pharmacol. (2014) 5:152. 10.3389/fphar.2014.00152 25076907 PMC4100575

[33] Garcia-Nicolas O Quereda JJ Gomez-Laguna J Salguero FJ Carrasco L Ramis G. Cytokines transcript levels in lung and lymphoid organs during genotype 1 Porcine Reproductive and Respiratory Syndrome Virus (PRRSV) infection. Vet Immunol Immunopathol. (2014) 160:26–40. 10.1016/j.vetimm.2014.03.008 24726859

[34] Weiss G Werner-Felmayer G Werner ER Grünewald K Wachter H Hentze MW. Iron regulates nitric oxide synthase activity by controlling nuclear transcription. J Exp Med. (1994) 180:969–76. 10.1084/jem.180.3.969 7520477 PMC2191642

[35] Käser T Gerner W Saalmüller A. Porcine regulatory T cells: Mechanisms and T-cell targets of suppression. Dev Compar Immunol. (2011) 35:1166–72. 10.1016/j.dci.2011.04.006 21530576

[36] Dantzer R Kelley KW. Twenty years of research on cytokine-induced sickness behavior. Brain Behav Immun. (2007) 21:153–60. 10.1016/j.bbi.2006.09.006 17088043 PMC1850954

[37] Georgieff MK. Iron assessment to protect the developing brain. Am J Clin Nutr. (2017) 106(Suppl 6):1588S−93S. 10.3945/ajcn.117.155846 29070550 PMC5701704

[38] Rytych JJL Elmore MRPM Burton MMD Conrad MMS Donovan SSM Dilger RNR. Early life iron deficiency impairs spatial cognition in neonatal piglets. J Nutr. (2012) 142:2050–6. 10.3945/jn.112.165522 23014488

